# Radiation Stricture of the Biliary Ducts: An Emerging New Entity?

**DOI:** 10.1155/1992/52618

**Published:** 1992

**Authors:** A. Halevy, A. Adam, G. Stamp, I. S. Benjamin, L. H. Blumgart

**Affiliations:** 1 Department of Surgery Assaf Harofeh Medical Center Zerifin 70300 Israel; 2 Department of Radiology Royal Postgraduate Medical School Hammersmith Hospital Du Cane Road London UK; 3 Department of Pathology Royal Postgraduate Medical School Hammersmith Hospital Du Cane Road London UK; 4 Department of Surgery Kings Hospital London UK; 5 Dept. of Surgery Memorial Sloan Kettering Cancer Centre 12175 York Av. New York New York 10021 USA

## Abstract

Two patients with stricture of the extrahepatic biliary tree are described. Both patients presented with a clinical picture of obstructive jaundice one to two years following radiotherapy for a malignant condition. As no recurrent tumour was detected in either of the patients the strictures were considered to be the result of radiation therapy. Bilio-enteric decompression was performed in both patients who are well at follow up one and ten years after the procedure.


HPB Surgery, 1992, Vol. 5, pp. 267–270
Reprints available directly from the publisher
Photocopying permitted by license only
© 1992 Harwood Academic Publishers GmbH
Printed in the United Kingdom

### A CASE REPORT

### RADIATION STRICTURE OF THE BILIARY DUCTS: AN EMERGING NEW ENTITY?

A. HALEVY[1], A. ADAM[2], G. STAMP[3], I.S. BENJAMIN[4] and L.H. BLUMGART[5]
*Department of Radiology[2] and Pathology[3], Royal Postgraduate Medical School, Hammersmith Hospital, Du Cane Road, London, UK*
*Department of Surgery[4], Kings Hospital, London, UK*

*(Received 17 June 1991)*

Two patients with stricture of the extrahepatic biliary tree are described. Both patients presented with a clinical picture of obstructive jaundice one to two years following radiotherapy for a malignant condition. As no recurrent tumour was detected in either of the patients the strictures were considered to be the result of radiation therapy. Bilio-enteric decompression was performed in both patients who are well at follow up one and ten years after the procedure.

KEY WORDS: Bile duct stricture, radiotherapy

### INTRODUCTION

Benign biliary strictures are, in the majority of cases, a result of iatrogenic damage to the biliary tree during cholecystectomy. Other causes for stricture such as inflammation, trauma, and pancreatitis are less common. Although strictures of the biliary system have been produced by irradiation in experimental models[1,2], we are aware of only one clinical case report of irradiation-induced stricture of the hepatic duct[3].

During a 14 year period the senior author (L.H.B.) has treated over 200 patients with benign biliary stricture, only two of whom presented with irradiation-induced stricture of the extrahepatic biliary tree. The following report presents these two cases, discusses the entity and suggests the treatment options.

### CASE REPORTS

### *Patient 1 — J.O. Male, Age 30 Years*

This patient was treated for Testicular Teratoma by surgery and radiotherapy one

[1]Present address: Department of Surgery, Assaf Harofeh Medical Center, Zerifin 70300, Israel

[5]Present address: Dept. of Surgery, Memorial Sloan Kettering Cancer Centre, 12175 York Av. New York, New York 10021

year prior to his presentation with obstructive jaundice. On presentation he was deeply jaundiced with bilirubin levels of 880 $\mu$mol/l. An ultrasound scan showed a dilated gall bladder and dilated intra- and extra-hepatic ducts. Percutaneous transhepatic cholangiography (PTC) showed a stricture at the lower end of the common bile duct in its retropancreatic portion.

Laparotomy was undertaken and no recurrent tumour was found during exploration. Cholecystectomy and a Roux-en-Y Choledochojejunostomy were performed. Biopsies taken from the site of the stricture were reported as showing inflammatory reaction and fibrosis. Ten years following surgery for decompression of his jaundice the patient is well.

*Patient 2 — R.B. Male, Age 63 Years*

This patient was treated during 1983 for Primary Hepatic Lymphoma by radiotherapy and chemotherapy. The patient presented two years later with a clinical picture of obstructive jaundice and cholangitis. An ultrasound scan showed dilated intrahepatic ducts and PTC demonstrated a stricture at the supraduodenal common bile duct (Figure 1). At operation the liver was found to be normal but there was gross thickening of the connective tissue and the nodal structures embracing the common bile duct in its supraduodenal and retroduodenal portion. Biopsies taken during surgery were reported to show dense hyalinised fibrous tissue with no malignancy. Cholecystectomy and Roux-en-Y Choledochojejunostomy were performed.

Histological examination of the gallbladder showed changes consistent with the effects of radiation. The postoperative course was uneventful. At follow up 2 $\frac{1}{2}$ years past surgery the patient was well. Shortly after his last attendance for follow up he was killed in an automobile accident in Portugal. An autopsy report is not available.

### DISCUSSION

Radiation hepatitis is a well described entity[4–5], the severity of which is dose-dependent. Although the effects of radiation on the liver have been extensively documented, little attention has been paid to the fate of the extra-hepatic biliary tree following irradiation to the liver and hepatic hilus. Todoroki[1], irradiating the hilus of the liver of rabbits by a single dose of 3000 rad electrons, found evidence of slight fibrosis, atrophy, and degeneration of the bile ducts. In a study in dogs, Sindelar *et al.*[2], showed that irradiation produced stricturing of the biliary duct. In fact, all dogs developed some form of mural thickening, fibrosis, atrophy of the mucosa and a degree of narrowing of the lumen of the common bile duct by six weeks following irradiation. The extent of the injury was dose-dependent. The clinical presentation of patients with irradiation-induced stricture of the extrahepatic biliary tree is that of obstructive jaundice. The essential diagnostic approach to such a patient follows the route of investigation for any patient with obstructive jaundice[9]. However, as these patients have been treated once for cancer, special emphasis should be given to the exclusion of local recurrence and/or metastatic spread as cause for the obstructive jaundice. It is of particular importance to note that jaundice is not necessarily due to advancing or recurrent malignancy in these patients, but the possibility of benign irradiation stricture should be considered.

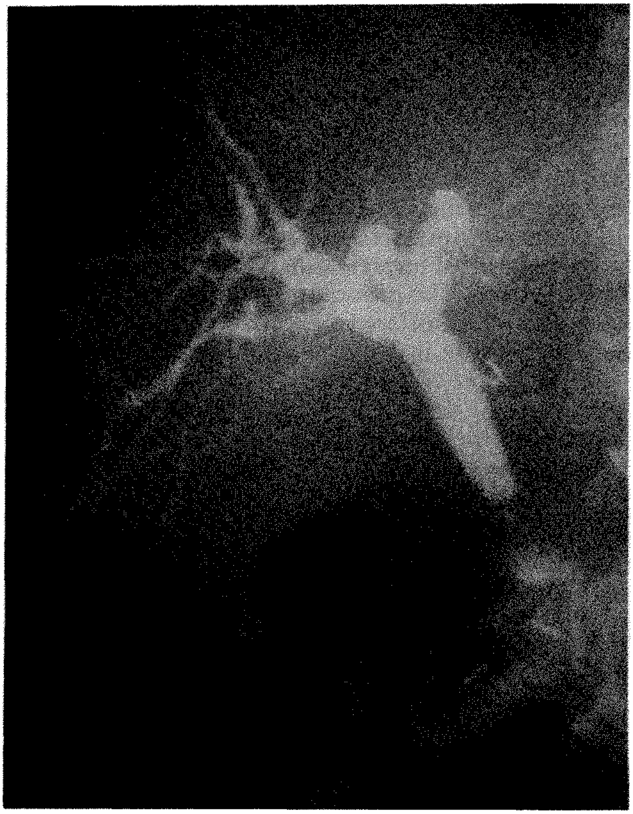

**Figure 1** PTC showing a stricture of the common bile duct. There is dilatation of the duct above the level of the stricture and some irregularity of the duct outline probably due to cholangitic changes.

The histological findings were characteristic of radiation changes including dense hyalinized collagen containing inflammatory cells with areas of marked collagen degeneration and intimal fibroblatic proliferation.

The surgical approach to these patients does not differ from the approach to any patient with benign biliary stricture. However, special emphasis must be given to biopsies taken from the area of stricture in order to exclude secondary spread of the original tumour. Both of our patients had a Roux-en-Y choledochojejunostomy[10]. The post-operative course was uneventful in both patients and the first patient is still fit and well more than ten years after bilio-enteric bypass.

Although neither of our patients had intra-operative radiotherapy, the risk of biliary stricture formation following such treatment may be similar to that following external beam therapy. Sindelar *et al.*[2], *in commenting on the results of their experimental study, suggest that clinical use of intraoperative radiation therapy of the bile ducts in humans may require routine use of biliary and duodenal bypass to prevent obstructive complications. We do not believe that a "preventive" bypass should be performed routinely and surgical intervention should be reserved only for that small number of patients who develop a stricture and jaundice. As radiation*

*therapy to the liver and hilar regions is used now more deliberately, there may be a rise in the number of patients presenting with radiation stricture of th eextrahepatic biliary tree.*

### SUMMARY

Stricture of the extrahepatic ducts as a result of radiotherapy to the liver and hilar region is rare but should be considered in patients developing obstructive jaundice following such therapy. Surgical intervention is required only in those patients who develop obstructive jaundice. The procedure of choice is a Roux-en-Y hepatico- or choledochojejunostomy, which offers an excellent long-term result. Biopsies must be taken from the stricture site in order to exclude secondary neoplastic spread.

### *References*

1. Todoroki, T. (1978) The late effects of single massive irradiation with electrons of the liver hilum of rabbits. *Jap. J. Gastroenterol. Surg.*, **11**, 169–177
2. Sindelar, W.F., Tepper, J. and Travis, E.L. (1982) Tolerance of bile duct to intra-operative irradiation. *Surgery*, **92**, 533–540
3. Chandrasekhara, K.L. and Iyer, S.K. (1984) Obstructive jaundice due to radiation induced hepatic duct stricture. *Am. J. Med.*, **77**, 723–724
4. Jeffrey, R.B., Moss, A.A., Quivey, J.M., Federle, M.D. and Wara, W.M. (1980) CT of radiation induced hepatic injury. *AJR*, **1315**, 445–448
5. Ingold, J.A., Reed, G.B., Kaplan, H.S. and Bagshaw, M.A. (1965) Radiation hepatitis. *Am. J. Roentgenol.*, **93**, 200–208
6. Lewin, K. and Millis, R.R. (1973) Human radiation hepatitis. A morphologic study with emphasis on the late changes. *Arch. Pathol.*, **96**, 21–26
7. Wharton, J.T., Delclos, L., Gallage, S. and Smith, J.P. (1973) Radiation hepatitis induced by abdominal irradiation with the cobalt 60 moving strip technique. *Am. J. Roentgenol.*, **117**, 73–80
8. Lansig, A.M., Davis, W.M. and Brizel, H.E. (1968) Radiation hepatitis. *Arch. Surg.*, **96**, 878–882
9. Bowley, N.B. and Benjamin, I.S. (1982) Diagnostic approach in the biliary tract. In: Blumgart L.H. (ed.) *The Biliary Tract, Clinical Surgery International*, Volume 5, London, Melbourne and New York; Churchill Livingstone, pp. 35–60
10. Voyles, C.R. and Blumgart, L.H. (1982) A technique for the construction of high biliary-enteric anastomosis. *Surg. Gynecol. Obstet.*, **154**, 885–887

(*Accepted by S. Bengmark 10 September 1991*))

